# The Promotion of Self-Regulated Learning (SRL) in the Classroom

**DOI:** 10.3390/bs16060856

**Published:** 2026-05-27

**Authors:** Stella Vosniadou

**Affiliations:** College of Education, Psychology and Social Work, Flinders University, Adelaide, SA 5042, Australia; stella.vosniadou@flinders.edu.au

## 1. Introduction

Self-regulated learning (SRL) refers to learners’ abilities to manage their learning by actively setting goals, monitoring their comprehension, controlling their emotion and motivation, and evaluating learning outcomes (Boekaerts & Corno, 2005; Hadwin et al., 2018; Pintrich, 2004; Zimmerman, 2002). Self-regulated learners can manage their attention, set goals, critically reflect on their learning, and maintain their motivation in the face of difficult tasks. On the contrary, non-self-regulated learners lack clear goals, depend on teachers and parents for structure and help, struggle with time management, have difficulty monitoring their comprehension, and give up easily when faced with challenges.

SRL has emerged in recent years as one of the most influential frameworks for understanding the skills and competencies needed to navigate today’s complex educational environments. Rapid technological changes and increasing reliance on digitally supported tools create opportunities for personalized learning while also placing great demands on students’ abilities to control their learning. Educational research has demonstrated that interventions that improve students’ metacognition and strategy use result in improved learning and student achievement; yet, research also shows that many students lack fundamental SRL competencies, particularly competencies related to comprehension monitoring, and have problems with persistence and sustained attention in less structured environments (Dignath & Büttner, 2008; Hattie et al., 1996; Bjork et al., 2013).

The development of SRL competencies is not an exclusively internal process; it is accomplished through participating in socially organized activities that include parents, teachers, peers, and cultural norms and practices. Educational environments and teachers, especially, can play an important role in developing students’ knowledge about SRL and in helping them acquire a powerful toolbox of learning strategies to manage their learning (Kramarski & Michalsky, 2009; Perry et al., 2015). However, no or little SRL instruction takes place in schools, and there are significant gaps in teachers’ beliefs, knowledge and instructional practices related to the implementation of SRL-supported pedagogy. Teacher education programs do not adequately prepare teachers to become effective facilitators of students’ SRL.

## 2. Research on Self-Regulated Learning

Considering the importance of developing learning environments that enable students to acquire the skills of a self-regulated learner, this Special Issue aims at presenting cutting-edge research that addresses a major problem in SRL research: How can the findings of SRL research be translated into effective classroom and school practices? The papers included in this Special Issue span several interconnected levels, ranging from socioeconomic differences in the use of SRL, teachers’ and students’ beliefs and misconceptions, students’ perceptions of teachers’ instruction, interventions to improve classroom learning environments, and the use of SRL research in schools. Several contributions also explore how SRL competencies can be empowered within online learning environments, emphasizing that SRL development is shaped by broader educational ecologies rather than by isolated learner characteristics alone.

In “Socioeconomic Differences in the Use of Self-Regulated Cognitive Strategies”, Guilia Raimondi and colleagues revealed a clear and consistent impact of SES on the use of cognitive strategies in the entire population of 10th grade students in Italy (Raimondi et al., 2026). Their research highlights the importance of the social and contextual dimensions of regulation and underscores the importance of educational interventions that help especially disadvantaged students develop their self-regulation competencies.

An important aspect of developing instruction that promotes SRL in the school context is to better understand relations between teachers’ and students’ SRL beliefs and their practices and instructional interventions that can influence them. Negative and inconsistent beliefs about learning and teaching and misconceptions about SRL have frequently been discussed in the literature as common barriers to the systematic and explicit instruction of self-regulation strategies (Vosniadou et al., 2021). In research with primary school teachers, Lies Backers and Hilde Van Keer investigated the alignment between the teachers’ knowledge and beliefs about SRL and their classroom practices (Backers & Van Keer, 2025). The study used video-based classroom observations combined with semi-structured interviews of eight teachers to capture both what teachers think and what they do. The findings revealed variation in SRL implementation, misalignments between knowledge, beliefs, and practice, misconceptions, limited self-efficacy, and school- and classroom-level factors constrained SRL implementation. The authors argued for the need for professional development addressing knowledge gaps, misconceptions, and teachers’ self-efficacy while encouraging school-wide reflective practices to support SRL in primary classrooms.

Also guided by research findings indicating the negative influence of learners’ inconsistent beliefs about SRL, both Fischer and Dignath and Skopeliti and colleagues explored ways to develop effective interventions that took into consideration students’ prior knowledge (Fischer & Dignath, 2026). Fischer and Dignath developed videos that modeled cognitive strategies while also addressing students’ beliefs. Their results confirmed the negative association between inconsistent beliefs and SRL use and the positive association between reported strategy use and SRL knowledge and self-efficacy in the 157 university students in their sample. Following the intervention, the participants, and especially those with low average SRL use, showed significantly greater increases in SRL knowledge, and reduced inconsistent beliefs. Along similar lines, Skopeliti and colleagues showed that the use of refutational texts and instructional analogies reduced conflicting beliefs about SRL in pre-service teachers (Skopeliti et al., 2025). Together, their findings suggest that targeted instructional interventions such as modeling videos, refutational texts, and instructional analogies can support the conceptual changes in learners’ beliefs about learning and teaching needed to support the development of SRL competencies.

Moving from individual students and teachers, the three articles that follow are all associated with the Teaching How to Learn (THL) project and investigated factors that contribute to the effectiveness of SRL instruction for secondary school students in Australia. In their article, Vosniadou and colleagues described the design, implementation, and evaluation of a professional development program that emphasized the combination of explicit strategy instruction with the design of learning activities that support student cognitive engagement and collaborative inquiry, encourage questioning and explanation, and provide metacognitive support (Vosniadou et al., 2025). Researchers have often linked the successful development of SRL skills in students with their involvement in meaningful and demanding tasks in constructivist learning environments. Yet, professional development courses usually focus exclusively on the explicit instruction of SRL and not on the broader classroom context, mainly because it has been difficult to find objective ways to measure the quality of the learning environment. Vosniadou and colleagues proposed that the Interactive, Constructive, Active, Passive (ICAP) theory (Chi, 2009) could be used as a basis for determining the cognitive engagement of students and that it provided an appropriate environment for the indirect promotion of SRL. They developed a framework and materials that helped teachers understand the distinction between the direct and indirect promotion of SRL and to change their instruction in ways that facilitated both. After the professional development course, the teachers increased the inclusion of interactive and constructive tasks in their lessons and promoted SRL directly through the inclusion of more motivational, metacognitive support statements, and explicit strategy promotion with reference to the benefits of SRL strategies.

Murdoch, Kang, White, and Graham examined another, overlooked aspect of SRL promotion: students’ perceptions and interpretations of teachers’ practices (Murdoch et al., 2025). The authors interviewed 25 students after they had watched videos of strategy instruction by teachers who had participated in the THL professional learning program mentioned earlier. The results revealed that both the teachers and the students focused on the teaching of subject content and the cognitive strategies required to process it. Furthermore, the results indicated that SRL instruction was most noticed by students when it consisted of the teachers naming the strategy, providing a clear process to be followed to apply the strategy, explaining how and why the strategy improves learning, and encouraging students to provide examples of how the strategy could transfer to other situations. The results highlight some of the critical aspects of SRL instruction that contribute to its effectiveness in promoting a greater range of strategies to students.

Extending the discussion beyond individual classrooms, Lawson and colleagues examined how SRL research findings were utilized within an Australian school (Lawson et al., 2025). The article provides details of how the teachers from one school used some of the research ideas central to the THL research project to further modify their teaching practices and influence school culture. The school program took place during a period of two years after the THL professional development course and demonstrated a systematic and innovative application of research ideas regarding the promotion of SRL at the school level. It consisted of three kinds of initiatives. The first involved individual teachers who decided to make more radical changes in their teaching, combining both changes in the learning environment through the adoption of more interactive and constructive tasks and explicit strategy instruction. The teachers involved took measures of student work at the start and end of the program that demonstrated the effectiveness of their teaching changes. The second initiative was a 6-week program of explicit instruction in six strategies for 12-year students taking certification exams, which was also associated with beneficial impacts on students’ external examination performance. The third was the formation of a student Learning Culture Group (LCG), which provided examples of how students can engage in the promotion of SRL strategies to other students. The LCG sought out resources, developed a program of discussions, invited attendance by other students, and designed and presented seminars on learning strategies specific to students who would enter Year 11 in 2023. Collectively, the three studies mentioned above demonstrate that the promotion of SRL requires coordinated efforts across multiple levels of the educational system, from teachers and students and classroom implementation to institutional uptake and systemic educational change.

Developments in digital and AI-supported learning environments have further expanded the possibilities of supporting SRL processes, while also highlighting the importance of self-regulation in the navigation of these environments. The remaining three contributions broaden the scope of this Special Issue from face-to-face instruction to instruction in digital environments. They address a critical issue in SRL promotion: how SRL skills can be facilitated in online and digital environments. Olga Aria-Gundin and colleagues examined the effectiveness of dynamic assessment to promote textual revision with 88 secondary school students (Arias-Gundin et al., 2026). The results showed that the students who received instruction on substantive text revision—focusing on deep, content-oriented revision—achieved the greatest gains compared to the students who were in a control group or received superficial, mechanical revisions. Kunyu Wang and colleagues, on the other hand, investigated how time management competencies can be strengthened in first-year university students through technology-enhanced experiential approaches (Wang et al., 2026). In their article they describe the development of a pedagogical model and a supporting digital tool that improved students’ planning, task completion, and time allocation and resulted in higher satisfaction with time use. In the last article, Jiahui Du, Lejia Liu, and Shikui Zhao investigated the development and implementation of a learning management system (LMS), which consisted of a conventional course content section and a training section designed to support students in applying SRL strategies (SRL-LMS) (Du et al., 2025). The findings showed that the university students who received the SRL-LMS reported more frequent and diverse application of SRL strategies and better academic performance in one of two exams compared to a control group that received only the content section of the LMS without the SRL support.

## 3. Conclusions

Taken together, the studies in this Special Issue reflect the growing maturity and expanding scope of SRL research. They illustrate the movement of the field from relatively narrow conceptions of strategy instruction toward richer understandings of learning as a dynamic, situated, socially mediated, and increasingly technology-supported process. Importantly, they also underscore the continuing need to bridge theory and practice by developing approaches to SRL promotion that are feasible, scalable, and responsive to the realities of contemporary classrooms.

As educational systems confront the demands of rapidly changing knowledge societies and AI-mediated learning environments, the promotion of SRL remains both an enduring challenge and an essential educational goal. Supporting students in becoming active, reflective, adaptive, and agentic learners is likely to remain central to educational research and practice in the years ahead. It is our hope that the contributions in this Special Issue will advance ongoing dialog regarding how classrooms can more effectively cultivate the regulatory capacities that learners need to thrive in increasingly complex educational and social worlds.
